# Molecular Modeling of Structures and Interaction of Human Corticotropin-Releasing Factor (CRF) Binding Protein and CRF Type-2 Receptor

**DOI:** 10.3389/fendo.2018.00043

**Published:** 2018-02-20

**Authors:** Paula G. Slater, Sebastian E. Gutierrez-Maldonado, Katia Gysling, Carlos F. Lagos

**Affiliations:** ^1^Department of Cellular and Molecular Biology, Faculty of Biological Sciences, Pontificia Universidad Católica de Chile, Santiago, Chile; ^2^Computational Biology Laboratory (DLab), Fundación Ciencia & Vida, Santiago, Chile; ^3^Department of Endocrinology, School of Medicine, Pontificia Universidad Católica de Chile, Santiago, Chile

**Keywords:** corticotropin-releasing factor, corticotropin-releasing factor binding protein, corticotropin-releasing factor receptor, class B G-protein coupled receptor, molecular modeling, molecular dynamics, protein-protein docking

## Abstract

The corticotropin-releasing factor (CRF) system is a key mediator of the stress response and addictive behavior. The CRF system includes four peptides: The CRF system includes four peptides: CRF, urocortins I–III, CRF binding protein (CRF-BP) that binds CRF with high affinity, and two class B G-protein coupled receptors CRF_1_R and CRF_2_R. CRF-BP is a secreted protein without significant sequence homology to CRF receptors or to any other known class of protein. Recently, it has been described a potentiation role of CRF-BP over CRF signaling through CRF_2_R in addictive-related neuronal plasticity and behavior. In addition, it has been described that CRF-BP is capable to physically interact specifically with the α isoform of CRF_2_R and acts like an escort protein increasing the amount of the receptor in the plasma membrane. At present, there are no available structures for CRF-BP or for full-length CRFR. Knowing and studying the structure of these proteins could be beneficial in order to characterize the CRF-BP/CRF_2α_R interaction. In this work, we report the modeling of CRF-BP and of full-length CRF_2α_R and CRF_2β_R based on the recently solved crystal structures of the transmembrane domains of the human glucagon receptor and human CRF_1_R, in addition with the resolved N-terminal extracellular domain of CRFRs. These models were further studied using molecular dynamics simulations and protein–protein docking. The results predicted a higher possibility of interaction of CRF-BP with CRF_2α_R than CRF_2β_R and yielded the possible residues conforming the interacting interface. Thus, the present study provides a framework for further investigation of the CRF-BP/CRF_2α_R interaction.

## Introduction

Corticotropin-releasing factor (CRF) system plays pivotal roles in the regulation of physiological responses and adaptation to stress (1, 2), and in the interaction between stress and addictive behavior (3). CRF activates the hypothalamic–pituitary–adrenal axis (4, 5) and also acts as neurotransmitter in different brain regions (2, 6).

The CRF peptides comprised CRF and urocortins I–III (UCNI–III), mediate their actions through the activation of two G-protein coupled receptors (GPCRs) CRF type-1 (CRF_1_R) and CRF type-2 (CRF_2_R). Although these receptors are encoded by different genes, they share a high sequence homology (70%) differing preferentially in their N-terminal domains (7, 8). Both receptors have splice variants. CRF_1_R has one functional and several non-functional isoforms and CRF_2_R has three functional isoforms in humans (α, β, and γ) that differ in their N-terminal domain and distribution, being the α variant the most abundant in the brain (6, 9).

CRF binding protein (CRF-BP), another CRF system member, is a protein with no significant sequence homology to that of CRF receptors (10) that binds CRF and UCNI with higher affinity than the receptors (11, 12). CRF-BP modulates CRF system actions (8, 9). An inhibitory role for CRF-BP was first described. CRF-BP is capable of binding most of the circulating CRF (13), influencing its half-life in human plasma (14) and inhibiting ACTH release in rat pituitary cells (10, 15). A facilitatory role for CRF-BP has also been described. CRF-BP facilitates CRF-dependent neuronal plasticity in the rat ventral tegmental area (VTA) (16) and stress-induced relapse to cocaine seeking behavior (17). These studies show that the facilitatory role of CRF-BP depends on CRF_2_R. In addition, it has been suggested that CRF-BP modulates ethanol binge drinking by a CRF_2_R-mediated mechanism (18).

We have recently shown that CRF-BP and CRF_2_R are co-expressed in a variety of VTA nerve terminals, including projections from the lateral hypothalamic area (19). In addition, we showed that CRF-BP physically interacts with CRF_2α_R in an isoform specific manner and that acts as CRF_2α_R escort-like protein facilitating the presence of the receptor in the plasma membrane (20). Thus, a deeper insight into the CRF-BP/CRF_2α_R interaction and determining the residues involved are the logical next steps on the study of the escort-like protein function and the facilitatory action of CRF-BP over CRF_2α_R.

CRF_2α_R belongs to class B1 subfamily of GPCRs. Obtaining the crystal structures of full-length class B GPCRs remains difficult because of technical issues regarding receptor production, purification, and stability (21, 22). Structures of the N-terminal extracellular domain (ECD) of various class B GPCRs have been determined by X-ray-crystallography and NMR (21) including CRF_2α_R (23) and CRF_2β_R (24, 25). The structure of the transmembrane domain (TM) of the human glucagon receptor (26) and CRF_1_R (27) have been reported, and more recently, the first structure of a full-length glucagon receptor in complex with an antibody and in its inactive conformation have been determined using X-ray-crystallography (28). On the other hand, there are no crystal structure or structural models reported for CRF-BP. The present study aimed to search for the prediction of the residues involved in the CRF-BP/CRF_2α_R interaction and the characterization of this interaction. Herein, we report the generation of comparative models of CRF-BP, CRF_2α_R, and CRF_2β_R (including the ECD and TM regions) and their analysis by means of molecular dynamics (MD) simulations and protein–protein docking.

## Materials and Methods

### Molecular Modeling of Human CRF, CRF-BP, CRF_2α_R, and CRF_2β_R

The molecular models of CRF-BP, CRF_2α_R, and CRF_2β_R were constructed using MODELER (29, 30), as implemented in the Protein Modeling module of Discovery Studio v2.1 (Accelrys Inc., San Diego, CA, USA). Human CRF-BP, CRF_2α_R, and CRF_2β_R reference sequences were retrieved from the Uniprot database, with accession numbers P24387, Q13324-1, and Q13324-2, respectively (31). CRF was modeled using the crystal structure of human CRF inactive analog (PDB: 1GO9) containing a D-Phe residue at position 12 and alpha-aminoisobutyric acid in position 15 (32).

For CRF-BP, top scoring models produced by threading-based approaches identified by Muster and Phyre2 servers (33, 34) were retrieved, aligned, and used as starting templates to generate a human CRF-BP model. Fragments from gastric intrinsic factor receptor cubilin (PDB: 3KQ4) (35) and neuropilin (PDB: 2QQL) (36) were used to construct the model. Secondary structure elements restraints such as α-helices and β-sheets as predicted by PCI-SS server (37) were included, as well as experimentally determined disulfide bridges (38) during modeling (Figure S1A in Supplementary Material).

For CRF receptors modeling, we used the crystal structure of CRF_1_R (PDB: 4K5Y) (27), the N-terminal ECD of human CRF_2α_R in complex with UCNI (PDB: 3N96) (23), and murine CRF_2β_R in complex with Astressin analog peptide (PDB: 2JND) (24) as templates. In addition, the crystal structure of the transmembrane bundle of glucagon receptor (PDB: 4L6R) was used as guide to model the N and C-terminal portions absent from the available CRF_1_R crystal structure (26) (Figures S1B,C in Supplementary Material). For each protein model, a set of 100 models were constructed and the best model according to Modeler internal PDF score was subjected to a molecular minimization protocol using the CHARMM22 force field available within Discovery Studio (39, 40). The protocol consisted of 5,000 steps of steepest descent method, followed by 10,000 steps of conjugate gradient method to reach a final root-mean-square (RMS) gradient of 0.001 kcal/mol/Å^2^.

The overall quality of the final models was assessed by Ramachandran plot using the RAMPAGE server and quality model assessment with ProSA (protein structure analysis) server, respectively (41, 42). ProSA web was used to check and compare the obtained protein structural models with those experimentally determined by X-ray crystallography or NMR (42). The ProSA *z*-score indicates overall model quality and measures the deviation of the total energy of the structure with respect to an energy distribution derived from random conformations. The APBS software was used to calculate the spatial distribution of electrostatic potential on protein atoms using a two-dielectric implicit solvent model and the finite difference method to solve the Poisson–Boltzmann Equation (43). The dielectric constant used was 4 for proteins and 80 for the solvent.

### MD Simulations

The CRF_2_Rs and the CRF_2α_R/CRF-BP complex were inserted into a 1-palmitoyl-2-oleoyl-sn-glycero-3-phosphocholine POPC lipid membrane considering the spatial arrangements of the protein with respect to the hydrocarbon core of the lipid bilayer, as obtained from the OPM database (44). For the CRF_2_R systems, a 150 Å × 150 Å × 120 Å box consisting of the protein, lipids, classic TIP3P model for water molecules, and 150 mM KCl was generated using the membrane builder module of CHARMM-GUI (45, 46). In a similar fashion, the CRF_2α_R/CRF-BP complex was embedded in a 140 Å × 140 Å × 160 Å box. MD simulations were carried out with the NAMD 2.9 simulation package (47), using the CHARMM36 force field parameters for proteins and lipids (48, 49). Periodic boundary conditions were imposed in all three directions and the Particle Mesh Ewald method was used to account for full long-range electrostatic interactions within the selected boundary condition within a relative tolerance of 1 × 10^−6^ (50). The final systems were composed of nearly 235,000 atoms for CRF_2_Rs, and nearly 255,000 atoms for the CRF_2α_R/CRF-BP complex. The simulations were started from different seeds, and three replicas of 100 ns for each CRF_2_ receptors were performed, while a single 100 ns simulation was performed for the CRF_2α_R/CRF-BP complex. A 12 Å cutoff was used to compute non-bonded interactions with a smooth switching function applied at a distance of 10 Å. To impose the thermal exchange with an external thermostat, the isobaric–isothermal ensemble (NPT) with constant number of particles N, pressure P, and temperature T was used. Constant temperature was maintained by coupling the system to a thermal bath whose temperature is maintained *via* Langevin dynamics with a friction coefficient of 1 ps^−1^. Constant pressure was maintained using a Langevin piston at a nominal value of 1 atm (51). The SHAKE algorithm, with a tolerance of 1 × 10^−8^ Å, was applied to constrain the length of all covalent bonds involving hydrogen, thus allowing the use of a 2 fs integration time step along with the r-RESPA integrator, which allows a multiple time step scheme where bonded, short-range non-bonded, and long-range electrostatic terms are calculated every 2, 2, and 4 fs, respectively. By plotting Cα-root-mean-square deviation (RMSD) and RMS fluctuation (RMSF) along the MD simulation, we assessed the structural equilibration reached by our models. To further characterize the structure of the CRF_2_Rs, three parameters were calculated: angle phi, defined between the hinge region (connecting the TM with the ECD) and the center of mass (COM) of the TM domain; angle theta, defined between the hinge region and the COM of the ECD; and the distance between the COM of both domains. Both angles helped define the orientation of the ECD with respect to the XY plane (parallel to the membrane plane) and the *Z* axis (perpendicular to the membrane) (52). Also, for the CRF_2α_R/CRF-BP complex, the total internal energy of the complex was calculated, as well as the total interaction energy between the CRF_2α_R and the CRF-BP in terms of its electrostatic and van der Waals components. These calculations were performed using the NAMD Energy analysis tool available in the Visual Molecular Dynamics v1.9.3 (VMD) software (53).

### Protein–Protein Docking and Protein–Protein Interactions (PPIs) Calculations

Protein–protein docking was performed using Hex v8.0 with default parameters (54). Briefly, for the generation of the top scoring solutions, we used an initial Steric Scan at *N* = 16, followed by a Final Search at *N* = 25, obtained by using just the steric contribution to the docking energy. We used the Shape only correlations, the 3D Fast Lite as FFT mode, with a grid dimension of 0.6 Å. These orientations are sorted by calculated energy, and a new set of trial orientations are generated for the top scoring. 10,000–20,000 orientations using the Scan Step and SubSteps were used to construct new distance samples in steps of ±(Scan Step 0.75 Å)/(Substeps 2) from the initial orientations, 1 Å resolution was used to scan the search space and a 0.5 Å resolution was used to perform the high-resolution scoring (55). A final minimization protocol for the top scoring solution complexes consisted of 20,000 steps of steepest descent method, followed by 10,000 steps of conjugate gradient method to reach a final RMS gradient of 0.001 kcal/mol/Å^2^ to obtain the final models. Protein interactions such as disulfide bonds, hydrophobic interactions, ionic interactions, hydrogen bonds, aromatic–aromatic interactions, aromatic–sulfur interactions, and cation–π interactions within a protein or between proteins in a complex were calculated using the PPI server (56).

## Results

### Corticotropin-Releasing Factor Binding Protein (CRF-BP) Modeling and Validation

**Figure 1:**
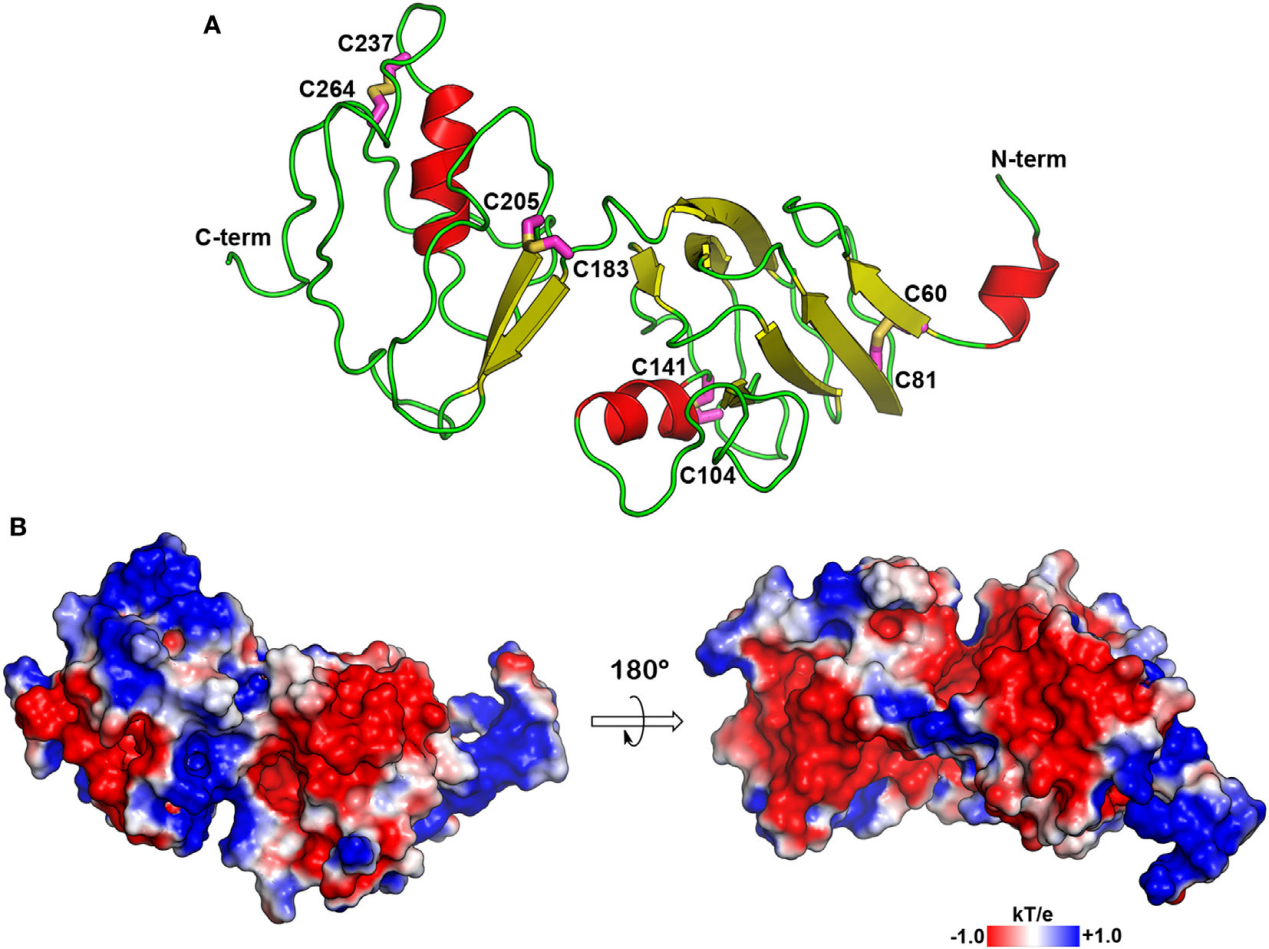
Molecular model of CRF-BP. **(A)** Secondary structure depiction of the obtained folding for human CRF-BP. The residues forming part of disulfide bridges are shown with the carbon atoms in magenta stick representation. **(B)** Electrostatic potential surface plotted onto the solvent accessible surface (±1 kT/e). The positive and negative electrostatic values are colored in blue and red, respectively.

We have previously demonstrated that CRF-BP and CRF_2α_R interact (20). In order to further characterize this interaction and predict which residues are forming the interacting interface, we reasoned that the prediction of structural models for CRF-BP and CRF_2_R were necessary. There are no crystal structures or modeling for CRF-BP. As CRF-BP sequence is conserved among species but displays no significant sequence similarity to any other known protein experimentally resolved; a threading approach was used to predict a model of the structure of CRF-BP. Fragments from neuropilin (PDB: 2QQL) (36) and gastric intrinsic factor receptor cubilin (PDB: 3KQ4) (35) were used as starting templates. The predicted structural model for CRF-BP fold consisted in two modules. The first module containing residues 50–180 which displayed two short alpha helices, six antiparallel beta-sheets, and the first pair of disulfide bridges C60–C81 and C104–C141. The second module comprised residues 180–245 of the protein, with a pair of beta-sheets and one alpha helix that contained a second pair of disulfide bridges C183–C205 and C237–C264 (Figure 1A). An electrostatic potential surface (EPS) was obtained for CRF-BP. In the protein, two acidic patches were observed (Figure 1B left, red colored) and when the protein was turned in 180°, two basic patches were observed (Figure 1B right, blue colored). In order to validate the predicted model, a Ramachandran plot distribution and a ProSA protein quality analysis were performed. Ramachandran statistics showed that more than 95% of the residues of the predicted model were in the allowed geometric regions for amino acids (Table 1; Figure S2A in Supplementary Material). Although some amino acids were positioned in the non-allowed regions, they were residues participating in protein turns. This result indicates that the obtained fold is feasible. ProSA protein quality analysis casted out a *Z*-score = −3.46 (Table 1), value that falls in the range of native structures (Figure S2B in Supplementary Material), indicating that a good quality protein model was predicted. The computational engine used for the calculation of *z*-score and plots uses knowledge-based potentials of mean force to evaluate model accuracy (57). The potentials of mean force compiled from the PDB database provide a statistical average over the known structures. A *z*-score within the range characteristic for native proteins is indicative of a correct structure (42).

**Table 1:** Protein modeling validation statistics.

Protein	Ramachandran plot analysis (% of residues)^a^			ProSA *Z*-score^b^
	Favored	Allowed	Outlier	
CRF-BP	91.1	4.9	4.0	−3.46
CRF_2α_R	97.1	1.8	1.1	−4.93
CRF_2β_R	95.9	2.7	1.4	−3.96

*^a^Calculated using the RAMPAGE server (http://mordred.bioc.cam.ac.uk/~rapper/rampage.php)*.

*^b^Calculated using the ProSA web server (https://prosa.services.came.sbg.ac.at/prosa.php)*.

**Figure 2:**
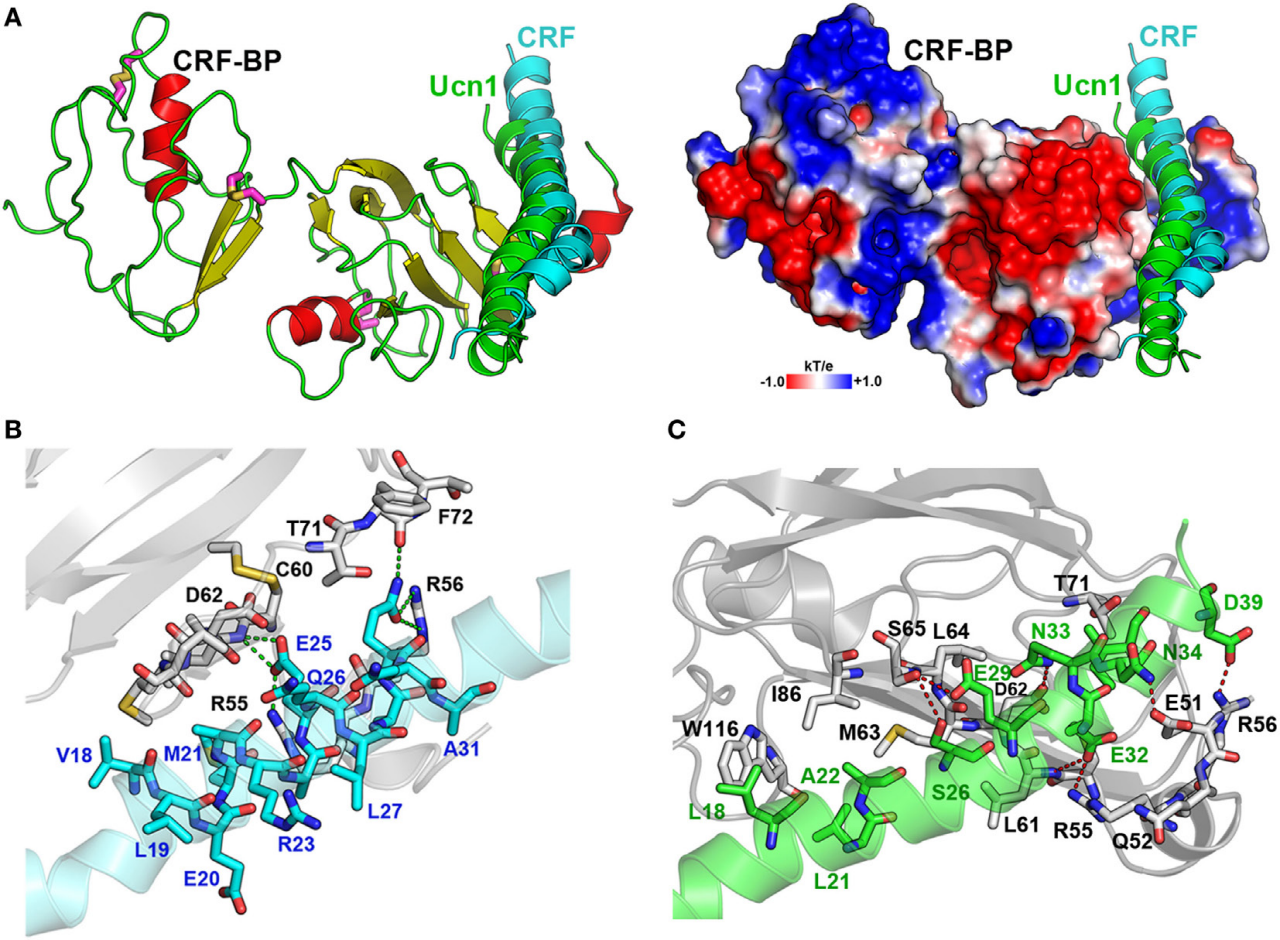
Protein-protein docking for CRF-BP with corticotropin-releasing factor (CRF) and UCNI. **(A)** Modeling of the interaction of CRF-BP with CRF and UCNI showing the secondary structure and the electrostatic potential surface plotted onto the solvent accessible surface of CRF-BP. **(B,C)** Magnifications of the interacting interfaces of CRF-BP with CRF **(B)** and CRF-BP with UCNI **(C)**. The interaction-important residues are shown with their carbon atoms in color, CRF-BP (white), CRF (cyan), and UCNI (green).

**Figure 3:**
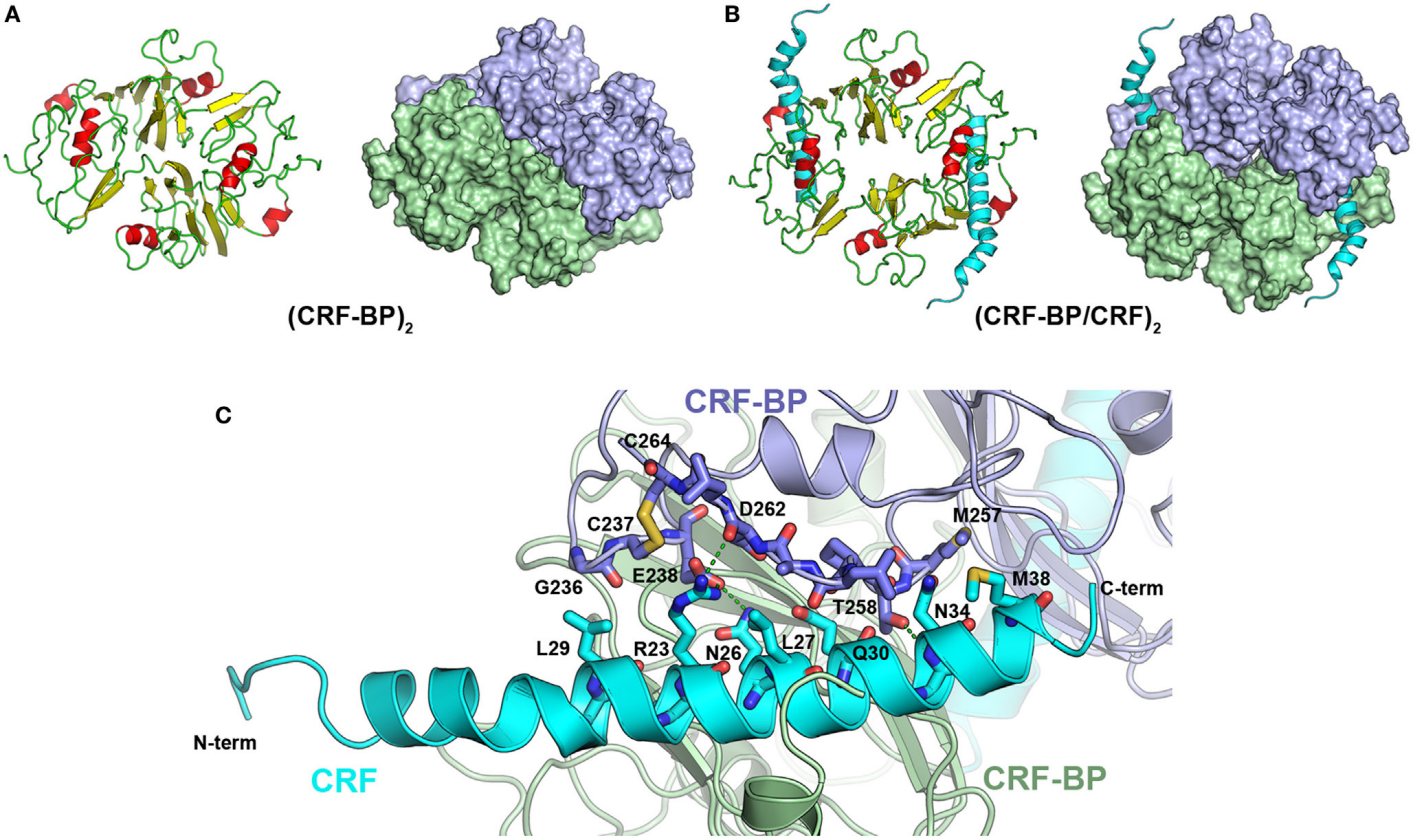
Protein–protein docking for CRF-BP homodimer. **(A)** Modeling of CRF-BP homodimer obtained from the protein–protein docking solution with lowest energy. **(B)** Modeling of CRF-BP homodimer interacting with two corticotropin-releasing factor (CRF). **(C)** Schematic representation of CRF-BP/CRF dimerization interaction of CRF and the C-terminal domain of CRF-BP.

CRF-BP is a protein that binds CRF and UCNI with high affinity, and these interactions have been well characterized (58, 59). To further validate the CRF-BP structural model, protein–protein docking experiments between CRF-BP with CRF and UCNI were performed and the predicted residues involved in this interaction were obtained and compared with previously published data. The obtained binding modes predicted that the C-terminal domain of CRF and UCNI may bind over the positively charged surface at the N-terminal domain of CRF-BP (Figure 2A). The CRF-BP/CRF interaction comprises mainly CRF-BP residues R55, R56, C60, L61, D62, M63, L64, T71, F72, and T73, and CRF residuesV18, M21, A22, E25, Q26, A28, and Q29 (Figure 2B). In addition, the CRF-BP/UCNI interaction comprises mainly CRF-BP E51, R55, R56, C60, L61, D62, M63, L64, S65, I86, and W116, and UCNI L18, L21, A22, S26, E29, E32, Q33, N34, I36, and D39 (Figure 2C). This obtained binding mode was in agreement with site-directed mutagenesis data from CRF-BP (12). Furthermore, it has been previously described that CRF-BP binds CRF as a dimer (59). Therefore, we also performed protein–protein docking experiments for two CRF-BP alone and with CRF in order to further validate our CRF-BP structural model. The results showed that the CRF-BP model was permissive for a symmetrical homodimerization arrangement (Figure 3A) and for interacting with CRF as a dimer (Figure 3B). Thus, all the aforementioned validation approach suggest that the predicted fold is feasible, of good quality, and in agreement to previously published data (12, 60). In addition, the obtained binding model for (CRF-BP/CRF)_2_ predicted that residues 19–38 of CRF are sandwiched by the CRF-BPs. In addition to already described interaction between CRF and a CRF-BP monomer, CRF may also interact with two patches within the C-terminal domain of CRF-BP (Figure 3C). CRF-BP residues 235–238 and 257–264 contacts CRF, with prediction of the residue E238 from CRF-BP displaying H-bond interactions with R23 and Q26 of CRF. CRF-BP D262 main chain carbonyl group also contact R23 of CRF. An additional H-bond interaction is predicted to occur between the side chain of T258 from CRF-BP and the main chain NH group of N34 from CRF.

### Corticotropin-Releasing Factor Type-2 Alpha (CRF_2a_R) and Type-2 Beta (CRF_2β_R) Receptors Modeling and Validation

**Figure 4:**
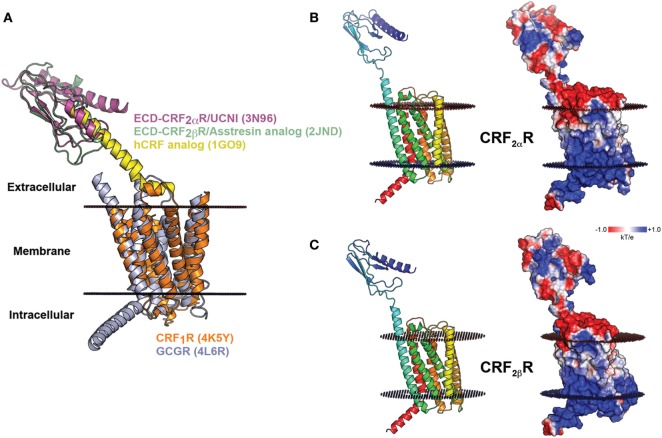
Molecular model of CRF_2α_R and CRF_2β_R. **(A)** Schematic representation of modeling of CRF_2_Rs generated based on the crystal structures of CRF_1_R and GCGR TM domains (orange and light blue, respectively) and the extracellular domain (ECD) of CRF_2α_R (magenta) and CRF_2β_R (light green) in complex with corticotropin-releasing factor (CRF) (yellow) analogs. **(B,C)**. Modeling of CRF_2α_R **(A)** and CRF_2β_R **(B)** and the electrostatic potential surface plotted onto the solvent accessible surface (± 1 kT/e) for both receptors. The positive and negative electrostatic values are colored blue and red, respectively.

There are still no full-length CRF receptor crystal structures available (21, 22). For CRF_2α_R and CRF_2β_R, only the ECD structures obtained by NMR are available, human CRF_2α_R-ECD in complex with UCNI, and murine CRF_2β_R-ECD in complex with an Astressin analog peptide (PDBs: 3N96 and 2JND, respectively) (23, 24). Thus, the recently solved crystal structure of CRF_1_R, in addition to the CRF_2α_R and CRF_2β_R ECDs available structures, were used as templates to predict a model for the structure of CRF_2_Rs (Figure 4A). The crystal structure of the transmembrane bundle from glucagon receptor (PDB: 4L6R) was used to model the extended helix 1 in the N-terminal region (TM1stalk region), the intracellular loop 2 (IC2), and the helix 8 in the C-terminal region, which are absent from the CRF_1_R crystal structure (21, 61). Considering the already available structural information and guided by similar works in class B GPCRs, the N-terminal domain of CRF was located in a position able to interact with the J-domain on the CRF_2_R TM bundle (62, 63). The EPS obtained for CRF_2_Rs showed that the electrostatic potential is similar for both receptors, with minor differences in the N-terminal region. The CRF_2β_R (Figure 4C) showed a more extensive basic patch than CRF_2α_R (Figure 4B).

**Figure 5:**
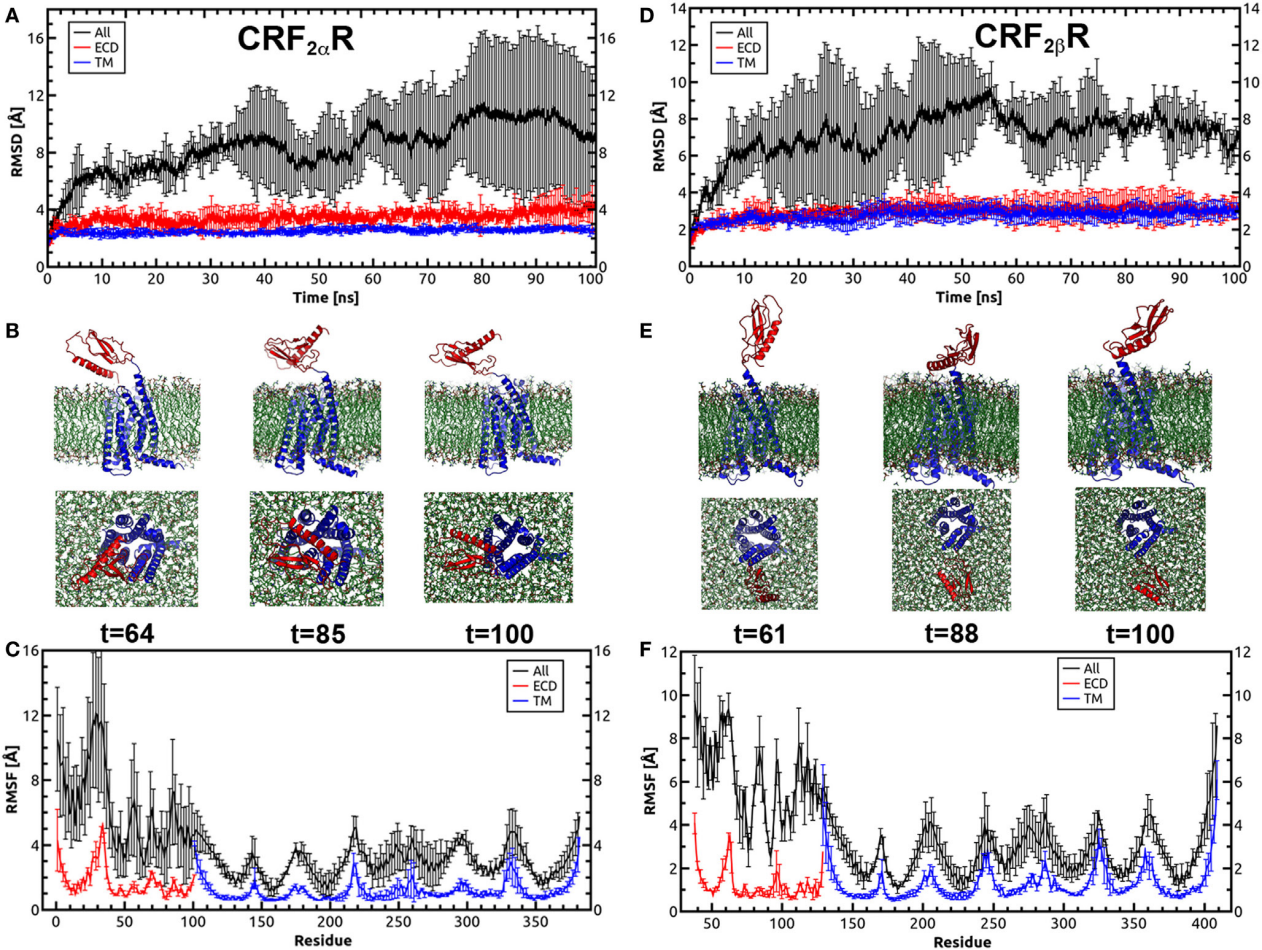
Molecular dynamics simulations for CRF_2α_R and CRF_2β_R. **(A,D)** Root-mean-square deviation (RMSD) values of the Cα atoms of CRF_2α_R **(A)** and CRF_2β_R **(D)** over the time course of the simulation. **(B,E)** Conformational states of CRF_2α_R **(B)** and CRF_2β_R **(E)** in the MD simulation showing the orientation of the extracellular domain (ECD) with respect to the TM domain. Three snapshots taken from specific periods of the MD simulation are shown from side and bottom views. **(C,F)** RMS fluctuation (RMSF) values of the Cα atoms of CRF_2α_R **(C)** and CRF_2β_R **(F)** residues. RMSD and RMSF values for the ECD (red lines), TM (blue lines), and full-length receptors (black lines) are shown.

Molecular dynamics were performed in order to test the stability of the CRF_2_R models including the ECD and TM regions. The receptors were embedded in a pre-equilibrated POPC lipid bilayer and solvated using the Membrane Builder in the CHARMM-GUI web server (Figure S3 in Supplementary Material). Each system was subjected to a 100 ns of MD simulations with three replicas. The RMSD and RMSF were computed over the course of the simulation for the Cα atoms of the proteins to measure structural stability and qualitatively characterize the dynamics of the proteins (Figure 5). The CRF_2α_R and CRF_2β_R MD trajectory analyses showed no significant changes in the RMSD values for the ECD (red lines) and TM (blue lines) regions (Figures 5A,D). However, the RMSD values calculated for the full-length receptors showed a significant change for CRF_2α_R and CRF_2β_R (Figures 5A,D, black lines). The changes obtained for CRF_2α_R can be attributed to translational and rotational movements of the ECD relative to the TM domain. The values obtained are coincident with three main different conformational states for CRF_2α_R: open-like, semi-closed, and closed-like (Figure 5B). The closed-like state was the one obtained at the end of the simulation, indicating that the receptor has a higher tendency for that conformation. In the case of CRF_2β_R, the values obtained are coincident with only one open-like conformational state, which varies the angle of extension (Figure 5E). RMSF describes the average fluctuation of each Cα atom of the amino acid residues in the proteins over the simulation time (Figures 5C,F). The general fluctuations of specific regions of the proteins are similar for both CRF_2_R. Within the TM region, the peaks of higher movement are coincident with the intra and extracellular loops. Moreover, in the ECD region, the peaks with higher movement are coincident with the loop that connects the α-helix with the β sheet bundle, and with the loop that connects the ECD with the TM region (stalk region).

**Figure 6:**
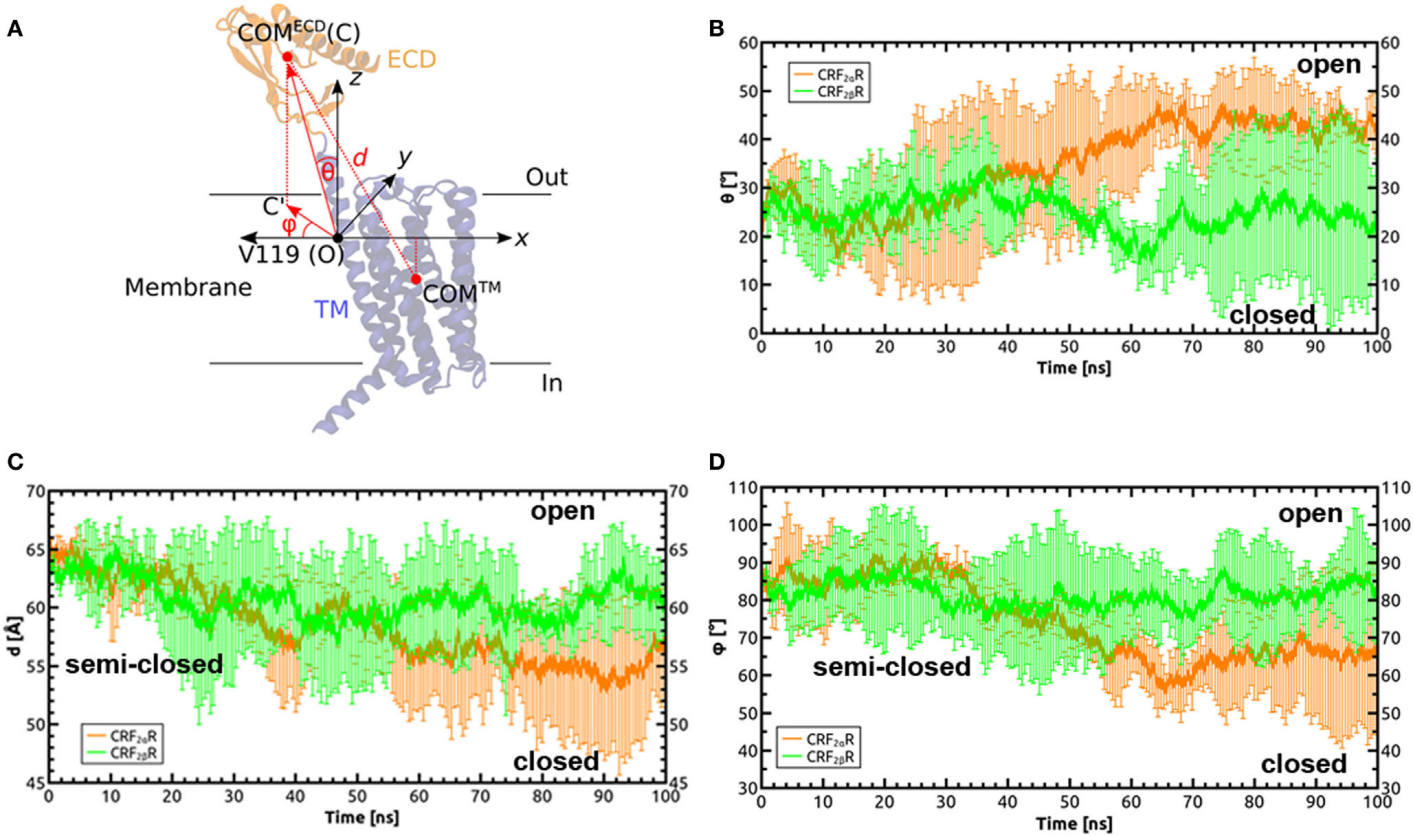
Defining orientation angles in the CRF_2α/β_R system. **(A)** Cartesian coordinate system defined to calculate the polar (θ) and Azimuthal (φ) angles, in order to define the relative orientations of the extracellular domain (ECD) with respect to the TM domain and the membrane plane during the molecular dynamics simulation of the receptors. The theta angle is defined as the angle between the vector formed by the origin (O) and the center of mass (COM) of the ECD **(C)**: $\vec{\text{OC}}$, with the *z* axis (perpendicular to the membrane plane). The phi angle is defined as the angle between the vector formed by the projection of the OC vector on the membrane plane ($\vec{\text{OC}’}$) and the *x* axis. Valine 119 in TM1 of the TM domain of CRF_2α_R was identified as the residue around which the helix bends to facilitate motions of the ECD. **(B–D)** Time dependences of θ, *d*, and ϕ in the MD simulations on CRF_2α_R (orange lines) and CRF_2β_R (green lines).

Both CRF_2α_R and CRF_2β_R showed great variation in phi and theta angles (Figure 6). Average theta angles for CRF_2α_R are near to the 40°–50° range, while for CRF_2β_R the average value is close to 20° ± 10° (Figure 6B). According to the data described for the GCGR, an angle of less than 20° corresponds to the closed state of the receptor while values close to 40° are associated to an opened state (52). Upon analyzing the phi angle, CRF_2α_R reaches an average value of 65°± 20°, while CRF_2β_ R stays at higher values at 85° ± 10° (Figure 6D). Analog to angle theta, low values of angle phi (~20°) have been associated with the closed conformation, which would mean that our simulations are either in the open conformation or in a semi-closed conformation. This is further supported by the distance between ECD and TM COMs, where both receptors reach similar values in the range of 55–60 Å (Figure 6C and Figure S4 in Supplementary Material), and this distance was associated with an open conformation. The results suggest that CRF_2β_R is more stable and displays mainly only one conformational state and that CRF_2α_R is less stable, reflected by more fluctuations within the Cα, and it has three main conformational states. In addition, in both receptors, the ECD region is the one with more fluctuations.

### Protein–Protein Docking for CRF-BP Binding to CRF_2_Rs

We previously demonstrated that CRF-BP physically interacts with the ECD of the α but not with the β CRF_2_R isoform (20). In order to test our models, a protein–protein docking was performed to predict the potential binding mode for CRF-BP with the ECD region of CRF_2_Rs. For the CRF-BP/CRF_2α_R interaction, characterization (20) immunofluorescence co-localization analyses using the Santa Cruz Biotechnology N-20 anti CRF_2_R antibody (residues W27-Q46 for CRF_2α_R and I53-Q73 for CRF_2β_R) were performed. We reasoned that, if the residues recognized by the antibody are available to bind the antibody they should not be participating on the CRF-BP/CRF_2α_R interaction, thus, these residues were excluded from the search space during the protein–protein docking assay. The best solution obtained for CRF-BP/CRF_2α_R docking casted out a total energy value of −838.1 kJ/mol and for CRF-BP/CRF_2β_R −685.9 kJ/mol (Table 2, Figure S5 in Supplementary Material). Both protein have similar number of residues (382 and 372) and molecular mass (44.53 and 43.74 kDa); therefore, the estimated binding energy values suggest that CRF-BP could bind to CRF_2α_R and form a more stable complex compared to CRF_2β_R.

**Table 2:** Protein–protein docking for CRBP binding to CRF2 subtype receptors.

CRF_2α_R-CRFBP			CRF_2β_R-CRFBP		
Cluster	Solution	Etotal (kJ/mol)	Cluster	Solution	Etotal (kJ/mol)
1	1	−838.1	1	1	−685.9
2	2	−827.2	2	2	−672.2
3	6	−783.2	3	4	−661.7
4	7	−756.6	4	5	−642.0
5	9	−735.6	5	6	−629.8
6	10	−734.4	6	7	−627.3
7	11	−702.4	7	8	−617.4
8	13	−700.9	8	9	−608.0
9	13	−698.9	9	10	−597.0
10	14	−698.1	10	11	−581.2

**Figure 7:**
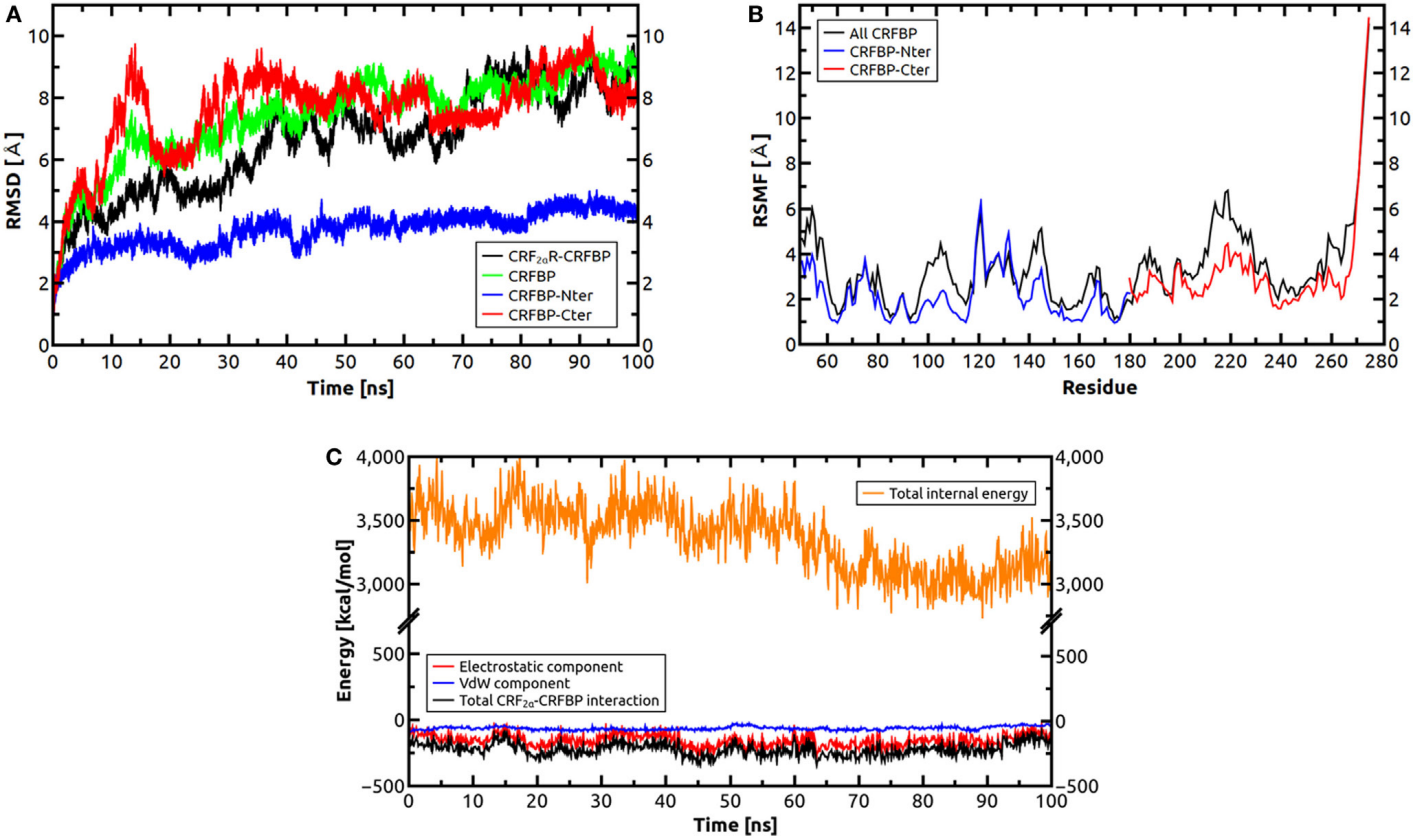
Molecular dynamics simulations for CRF_2α_R/CRF-BP complex. **(A)** Root-mean-square deviation (RMSD) values of the Cα atoms of CRF_2α_R, CRF_2α_R/CRF-BP, and CRF-BP N- and C-terminal domains. RMSD values of Cα atoms of CRF_2α_R/CRF-BP (black line), CRF-BP only (green line), and CRF-BP N- and C-terminal domains (blue and red lines, respectively) are shown. **(B)** RMS fluctuation (RMSF) values of the Cα atoms of CRF-BP. RMSF values for the N-term (blue lines), C-term (red lines), and full-length CRF-BP (black lines) are shown. **(C)** Interaction energy of the CRF_2α_R/CRF-BP complex.

**Figure 8:**
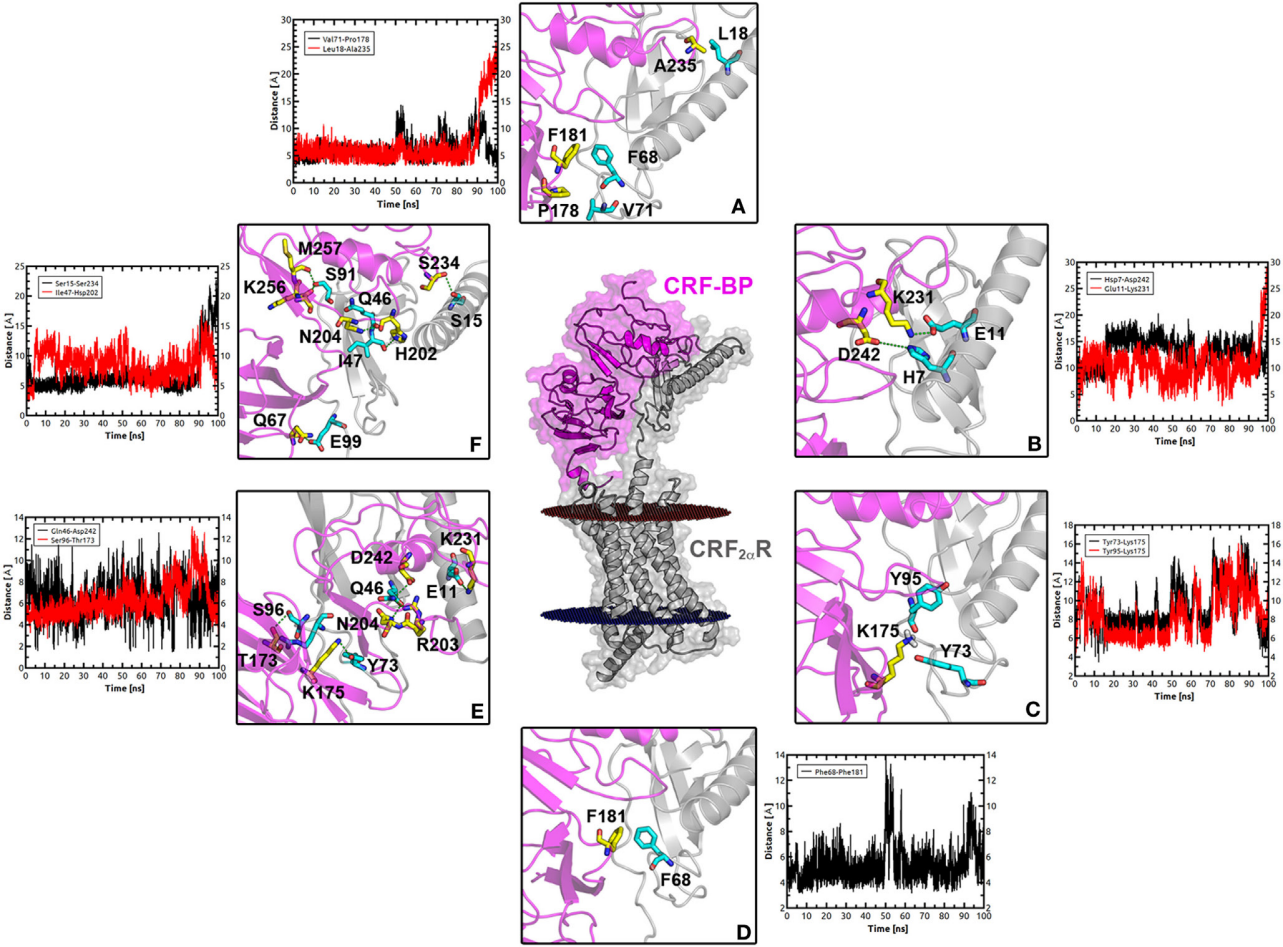
Interacting interface between CRF-BP and CRF_2α_R. **(A–F)** Magnifications of the interacting interface of CRF-BP with CRF_2α_R, showing different kinds of interactions determined using the protein interaction server. Hydrophobic interactions **(A)**, ionic interactions within 6 Å **(B)**, cation–π interaction within 6 Å **(C)**, aromatic-aromatic interactions within 4.5–7 Å **(D)**, protein–protein side chain hydrogen bonds **(E)**, and protein–protein main chain hydrogen bonds **(F)**. The measurement of these interactions was followed during a 100 ns molecular dynamics simulation of the CRF-BP/CRF_2α_R predicted complex.

Molecular dynamics simulations were performed to test the stability of the predicted CRF-BP-CRF_2α_R model and qualitatively characterize the dynamics of the complex (Figure 7). The CRF-BP-CRF_2α_R complex MD trajectory analyses showed no significant changes in the RMSD values for the CRF-BP N-terminal domain (blue lines). However, the RMSD values calculated for the full-length complex, CRF-BP and CRF-BP C-terminal domain display a significant variation (Figure 7A green, black, and red lines, respectively). The changes can be attributed to translational and rotational movements of the ECD relative to the TM domain and particularly the C-terminal domain of CRF-BP, in agreement to the higher RMSF of the C-terminal domain of CRF-BP (Figure 7B). The energy of interaction of the CRF-BP-CRF_2α_R complex during the dynamics indicates that the complex is stable and that the main contribution comes from electrostatics rather than van der Waals interactions (Figure 7C). This phenomenon was observed along with loss of connections between C-terminal domain of CRF-BP and CRF_2α_R, as shown by enhanced flexibility with respect to starting conformation. The interaction between CRF-BP and CRF_2α_R, the amino acids present in the interacting interface previously determined using the Protein interaction server (56), were also measured during the CRF-BP-CRF_2α_R complex dynamics. For CRF-BP/CRF_2α_R interaction interface, hydrophobic interactions (Figure 8A), ionic interactions within 6 Å (Figure 8B), cation–π interaction within 6 Å (Figure 8C), aromatic–aromatic interactions within 4.5–7 Å (Figure 8D), protein–protein side chain hydrogen bonds (Figure 8E), and protein–protein main chain hydrogen bonds were characterized (Figure 8F). Interactions remain in a similar range through most part of the dynamics upon loss of contacts in the last part of the simulation time, in agreement with a higher RMSF of this zone in CRF-BP.

In order to explore the relative contribution of these residues in the binding affinity for CRF-BP/CRF_2α_R, different point mutations of CRF_2α_R, and mutant combinations, were generated and the binding energy values were determined and compared with the WT (Table 3). The deletion of the first 12 amino acids (12aa) has a high contribution to the loss of the binding energy values predicted, and the loss was even higher in the 12aa/Y95Q/S96G/Q97E mutants. These results suggest that the first 12aa, that conform the α-helix, are important contributors and have synergistically effects with Y95, S96, and Q97 for CRF-BP/CRF_2α_R binding affinity.

**Table 3:** Protein–protein docking energies of CRF-BP to CRF_2α_R mutants.

Protein	Mutant residues								Etotal (kJ/mol)
	H7	E11	F68	V71	Y73	Y95	S96	Q97	
WT									−657.1
MUT 1	D	K							−627.3
MUT 2.1						L	V	L	−686.6
MUT 2.2						L	G	L	−690.7
MUT 2.3						L	V	E	−690.7
MUT 2.4						L	G	E	−660.5
MUT 2.5						Q	V	L	−700.6
MUT 2.6						Q	G	L	−700.6
MUT 2.7						Q	V	E	−660.7
MUT 2.8						Q	G	E	−639.2
MUT 3.1			N	S	L				−655.3
MUT 3.2			N	S	Q				−641.8
MUT 3.3			L	S	L				−705.9
MUT 3.4			L	S	Q				−629.9
MUT 4	D	K				Q	G	E	−655.5
MUT 5	D	K	L	S	Q				−623.9
MUT 6			L	S	Q	Q	G	E	−651.4
MUT 7	D	K	L	S	Q	Q	G	E	−623.1
									
	Deletion		F68	V71	Y73	Y95	S96	Q97	
MUT 8	1–12								−580.0
MUT 9	1–12					Q	G	E	−566.2
MUT 10	1–12		L	S	Q				−615.7
MUT 11	1–12		L	S	Q	Q	G	E	−581.2

## Discussion

In the present study, we predicted and validated the structural models for CRF-BP, CRF_2α_R, and CRF_2β_R. These models could be used for future investigation in order to further explore the CRF system. In addition, we were able to predict a higher possibility of interaction of CRF-BP with CRF_2α_R than CRF_2β_R. Even more, we predicted the residues that could be participating in the CRF-BP/CRF_2α_R interaction.

The predicted CRF-BP model displayed good Ramachandran plot distribution and ProSA protein quality assay. In addition, the predicted binding modes of CRF-BP with CRF and CRF-BP with UNCI are consistent with published site-directed mutagenesis data and functional assays showing pivotal roles for CRF-BP R56 and D62 in the interaction with CRF and R56, M63, and L64 in the interaction with UCNI (12). Even more, the obtained CRF-BP model was permissive for homodimerization and for interacting with CRF as a dimer, which is also in agreement with previously published data indicating that the CRF-BP generates a dimer form complex after binding to CRF (64). Furthermore, CRF-BP contains an alpha helix in the N-terminal region determined as the sorting signal for CRF-BP to entering the regulated secretory pathway (*Unpublished results*), the obtained CRF-BP model also presents this secondary structure. Even though we were able to obtain low-accuracy model (65) for CRF-BP because it shares less than 30% of sequence homology with the protein fragments used as templates, the aforementioned data all together demonstrated that the obtained CRF-BP model is feasible, of good quality and in agreement to previously published data. Thus, the model is suitable for further structural predictions and modeling of PPIs.

For CRF_2_Rs, we were able to obtain a high-accuracy model due to a high sequence homology with the template structures (65). Models were obtained using a comparative modeling approach and the crystal transmembrane structure of CRF_1_R (27) that shared a 70% sequence homology with CRF_2_Rs (9), and to N-terminal ECD of human CRF_2α_R (23), and murine CRF_2β_R (24) obtained by NMR as templates.

The accuracy of the model is important to define the predictions and studies that can be performed with them. As our models are not obtained by NMR or X-ray, that could achieve even a 100% accuracy, limitations in their use to study catalytic mechanisms, and designing and improving ligands are needed to be considered, although, our models can be used in studies including, docking of small ligands, defining antibody epitopes, refining NMR structures, among others (65).

The RMSD values obtained for CRF_2α_R showed movements of the ECD related to the TM domain and suggest three different conformational states. Similar results have been observed for the glucagon receptor (52, 63), which are consistent with the behavior of the two domain model described for class B GPCRs (66).

Considering the conformational states obtained for the CRF_2_Rs in the MD, the higher tendency for CRF_2α_R and CRF_2β_R is to be in the closed-like and semi-closed conformation, respectively. The differences could be explained by the differences in length and composition in the CRF_2α_R and CRF_2β_R N-terminal domain. It has been described that CRF_2α_R and CRF_2β_R differ in their N-terminal domain, due to alternative splicing; the first 34 amino acids of the CRF_2_R α isoform are replaced by 54 different amino acids in the β isoform (67). In addition, the β isoform has a cleavable signal peptide while the α isoform has a non-cleavable pseudo signal peptide, resulting in the absence and presence of the N-terminal α-helix, respectively (68, 69). Considering that CRF_2α_R is localized mainly intracellularly (70–73) and CRF-BP binds the N-terminal domain of CRF_2α_R and acts as an escort protein increasing the levels of the receptor in the plasma membrane (20), an open-like conformational state tendency for CRF_2α_R should be expected in order to be able to interact with CRF-BP. Even more, the CRF-BP/CRF_2α_R MD suggests that CRF-BP stabilizes the receptor in the open-like state.

The protein–protein docking performed between CRF-BP with CRF_2α_R, and CRF-BP with CRF_2β_R showed higher affinity of CRF-BP for CRF_2α_R than CRF_2β_R, this is in agreement with our previous results showing an isoform specific interaction between CRF-BP and CRF_2α_R (20). MD simulations of the predicted binding mode of CRF-BP to CRF_2α_R indicates that interactions remain in a similar range through most part of the dynamics upon loss of contacts in the last part of the simulation time, in agreement with a higher RMSF of this zone in CRF-BP. This phenomenon was observed along with loss of connections between C-terminal domain of CRF-BP and CRF_2α_R, as shown by enhanced flexibility with respect to starting conformation.

In addition, considering the aforementioned differences between CRF_2α_R and CRF_2β_R in their N-terminal region and the EPS obtained for both CRF_2_R isoforms showing different charge patches in their N-terminal region it makes sense to predict an isoform specific CRF-BP/CRF_2_R interaction dependent on the first 12aa in the N-terminal region. Even more, CRF-BP is a CRF_2α_R escort protein, and there is evidence showing that the escort proteins RAMP1-3 bind the α-helix of the calcitonin receptor (74), further supporting the idea of CRF-BP binding the first amino acids, which conforms the α-helix of the CRF_2α_R. Although, experimental approaches will be necessary to confirm the interacting interface.

It has been described that CRF-dependent neuronal plasticity in the VTA and stress-induced relapse to cocaine seeking behavior is dependent on CRF-BP and CRF_2_R (16, 17). It would be interesting to determine if the interaction between CRF-BP and CRF_2α_R is necessary for these CRF-dependent effects. The structural models generated in the present study could be used for the design of specific peptides capable of blocking the CRF-BP/CRF_2α_R interaction and test the implication of this interaction on the CRF-dependent effects.

There is a large fraction of sequences whose structure is difficult to be determined experimentally, like GPCRs, thus, structure prediction is important to obtain structural information. In this regard, the models reported herein provide a structural framework to work on further hypotheses and open new avenues of research on the CRF system.

## Conclusion

In summary, our results provide the first molecular models for CRF-BP and for full-length CRF_2α_R and CRF_2β_R. These molecular models allowed predicting the residues involved in the CRF-BP/CRF_2α_R interaction. These results are the starting point for future studies of the effect of the CRF-BP/CRF_2α_R interaction on stress-induced relapse to drug seeking behavior.

## Author Contributions

PS, KG, and CL conceived and designed research; CL and SG-M performed molecular modeling and simulations; PS, SG-M, CL, and KG analyzed data; PS, KG, and CL wrote the paper. All authors approved the final version of the manuscript.

## Conflict of Interest Statement

The authors declare that the research was conducted in the absence of any commercial or financial relationships that could be construed as a potential conflict of interest.

## Supplementary Material

Data Sheet S2 PDB files coordinates for all proteins.
