# Supplementary material for: Molecular Modeling of Structures and Interaction of Human Corticotropin-Releasing Factor (CRF) Binding Protein and CRF Type-2 Receptor
Source: Front Endocrinol (Lausanne). 2018 Feb 20;9:43. doi: 10.3389/fendo.2018.00043 (PMC5826306; doi:10.3389/fendo.2018.00043)
Supplement: Supplementary file 1 [file Data_Sheet_1.zip › Slater et al 2018_SI.docx]

**Molecular Modeling of Structures and Interaction of Human Corticotropin-Releasing Factor (CRF) Binding Protein and CRF Type-2 Receptor**

**Paula G. Slater^1,†^, Sebastian E. Gutierrez-Maldonado^2,†^, Katia Gysling^1,*^, Carlos F. Lagos^3,*^**

^1^Department of Cellular and Molecular Biology, Faculty of Biological Sciences, Pontificia Universidad Católica de Chile, Alameda 340, Santiago, Chile.

^2^Computational Biology Lab (DLab), Fundación Ciencia y Vida, Zañartu 1482, Ñuñoa, Santiago, Chile.

^3^Department of Endocrinology, School of Medicine, Pontificia Universidad Católica de Chile, Lira 85, 5th Floor, Santiago, Chile.

*** Correspondence:**Katia Gysling
[kgysling@bio.puc.cl](mailto:kgysling@bio.puc.cl)
Carlos F. Lagos
[cflagos@uc.cl](mailto:cflagos@uc.cl)

† Both authors contributed equally

**Supplementary Material**

**a) Supplementary Figure 1. Sequence alignments used for modeling CRF-BP, CRF_2α_R and CRF_2β_R**

**b) Supplementary Figure 2. Ramachandran & Z-score plots for CRF-BP, CRF_2α_R and CRF_2β_R models.**

**c) Supplementary Figure 3. Schematic representation of CRF_2_Rs systems for molecular dynamics.**

**d) Supplementary Figure 4. All runs angles and distances for CRF_2α_R and CRF_2β_R during molecular dynamics.**

**e) Supplementary Figure 5. Schematic representation of top binding clusters solutions of CRF-BP protein-protein docking to CRF_2α_R.**

**e) PDB files of minimized models of CRF-BP, CRF_2α_R and CRF_2β_R.**

**q**

**A**

**
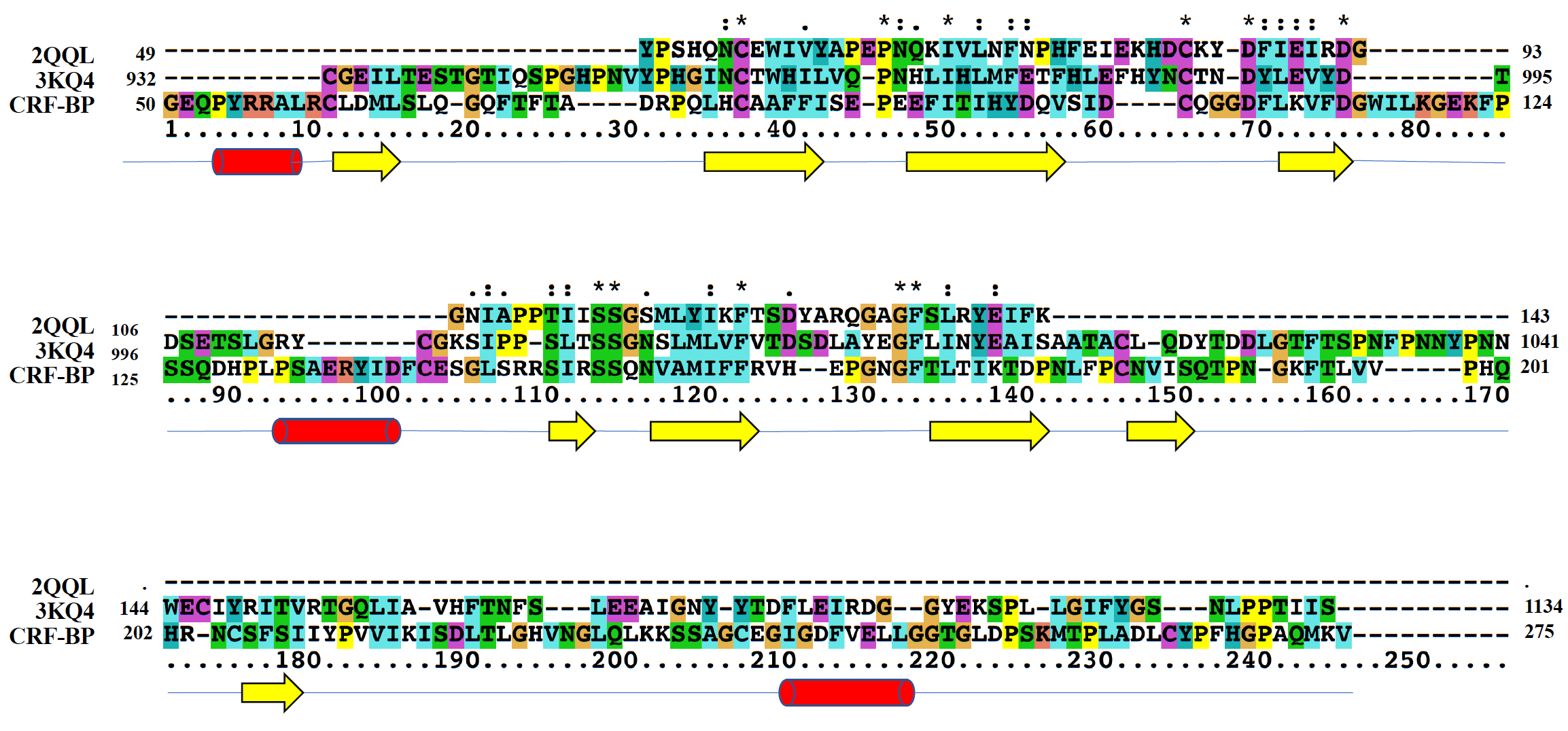
**

**B**

**
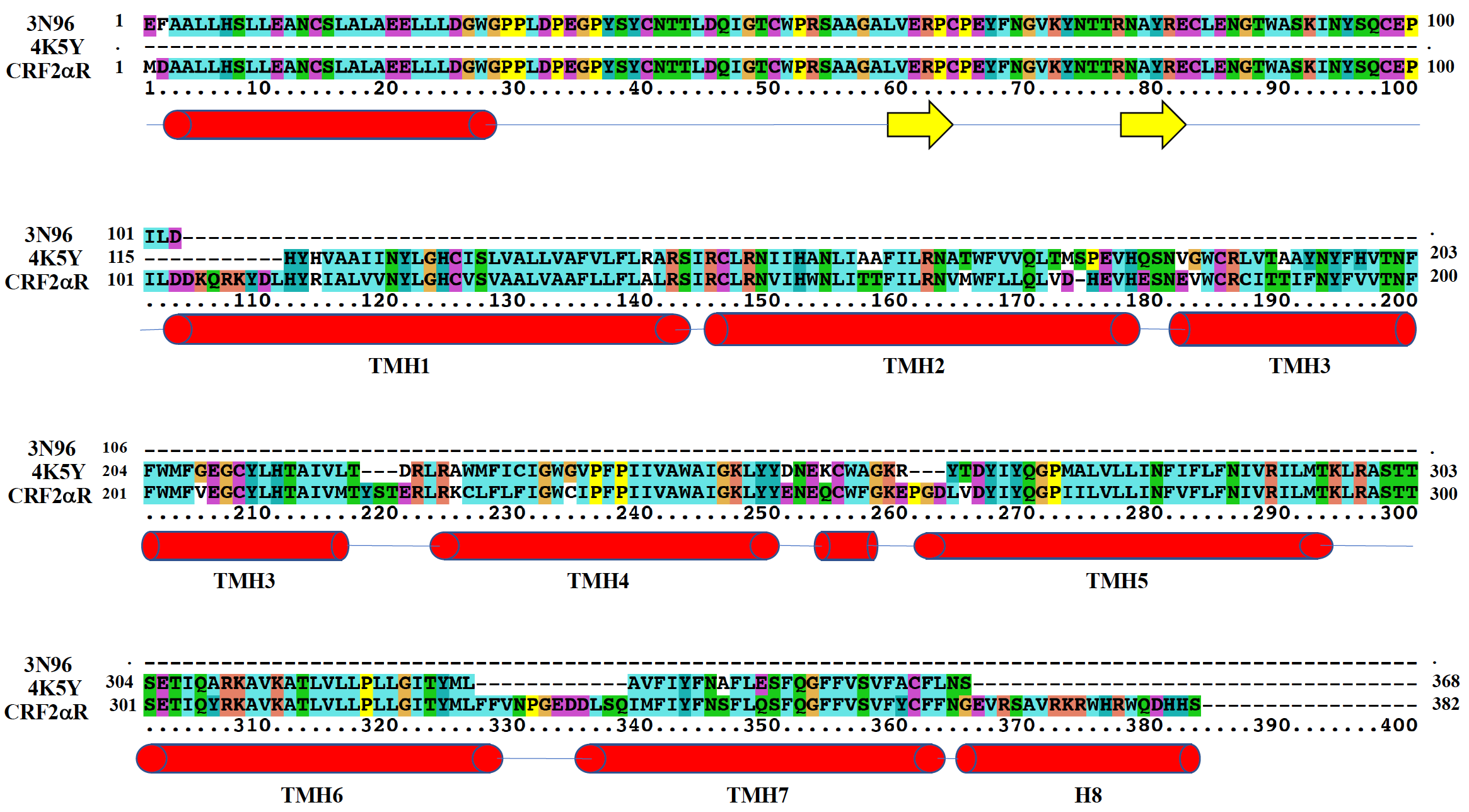
**

**C**

**
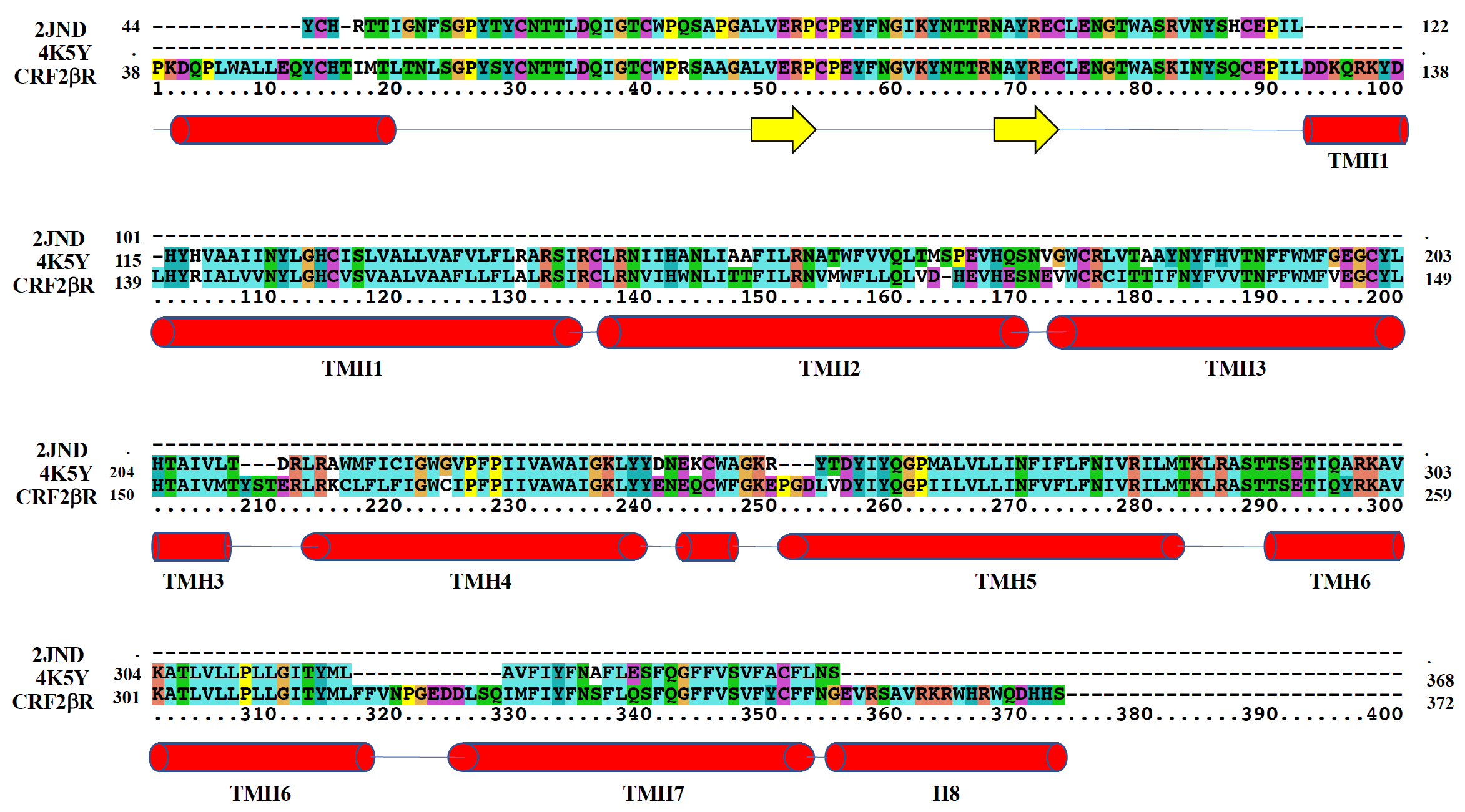
**

**Supplementary Figure 1. Sequence alignments used for comparative modeling of A) CRF-BP, B) CRF_2α_R and C) CRF_2β_R. Secondary structure elements are shown as red sticks (alpha helices) or yellow arrows (beta-strands).**

**
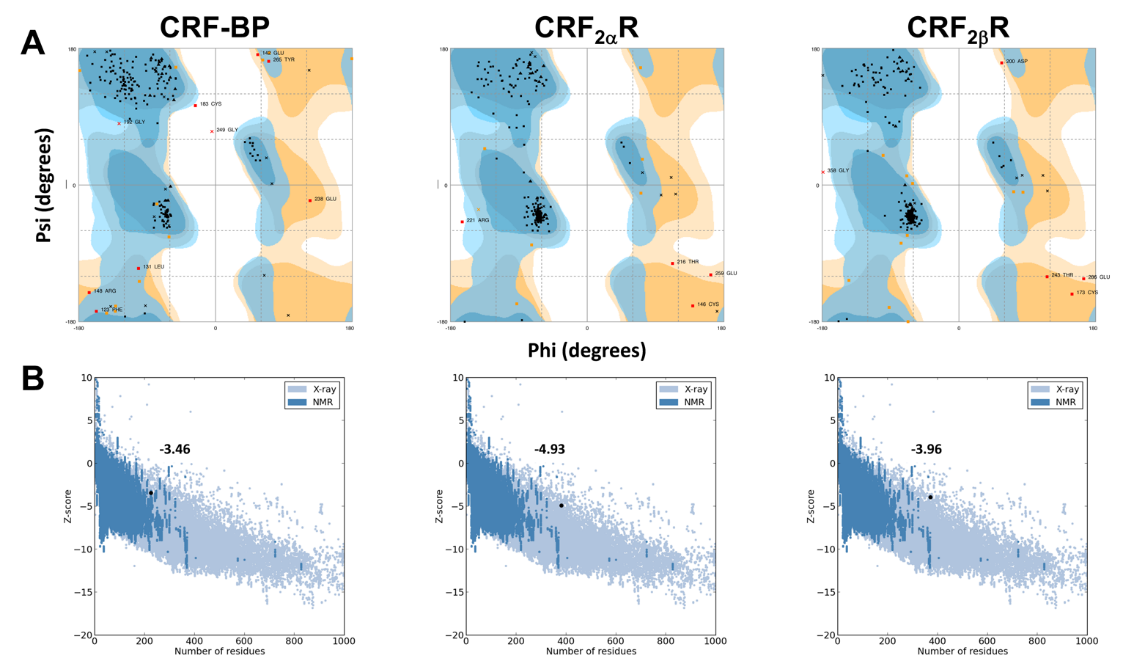
**

**Supplementary Figure 2. Ramachandran & Z-score plots for CRF-BP, CRF_2α_R and CRF_2β_R models. A)** Ramachandran plots for CRF-BP, CRF_2α_R and CRF_2β_R models. **B)** ProSA z-score for CRF-BP, CRF_2α_R and CRF_2β_R models, the contains the z-scores of all experimentally determined protein chains in current PDB. Groups of structures from different sources (X-ray, NMR) are distinguished by different colors. The z-score of the modeled proteins are within the range of scores typically found for proteins of similar size belonging to one of these groups (1).

**
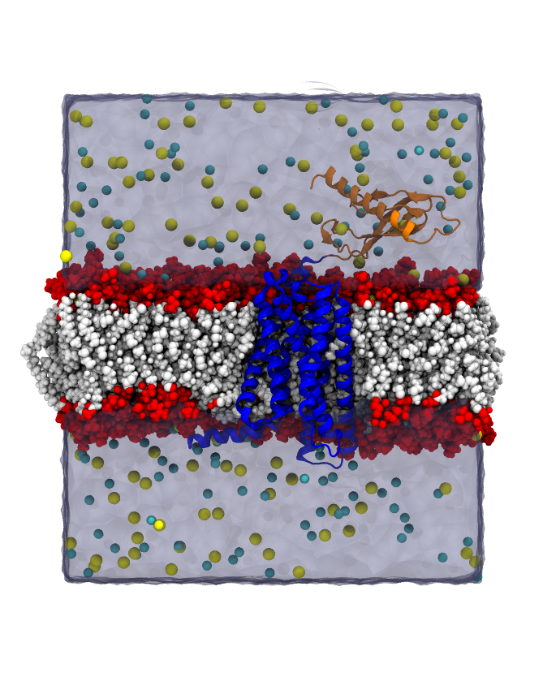
**

**Supplementary Figure 3. Schematic representation of CRF_2_Rs systems for molecular dynamics**

The CRF_2_Rs were inserted into a lipid membrane considering the spatial arrangements of the protein with respect to the hydrocarbon core of the lipid bilayer. For the CRF_2_R systems, a 150×150×120 Å box consisting of the protein, lipids, water molecules and 150 mM KCl was generated using the membrane builder module of CHARMM-GUI (2, 3).

**
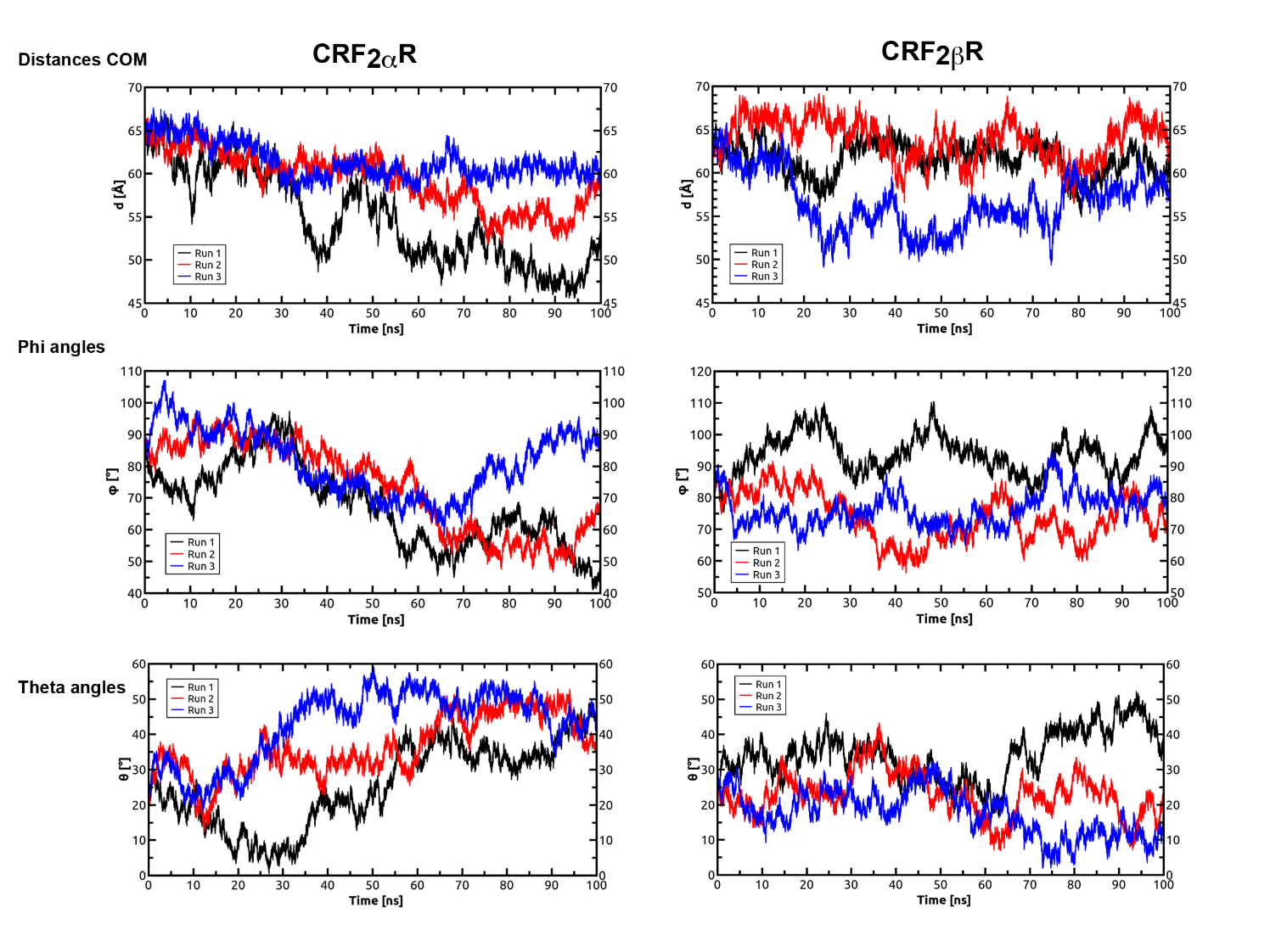
**

**Supplementary Figure 4. All runs angles and distances for CRF_2α_R and CRF_2β_R during molecular dynamics.**

**
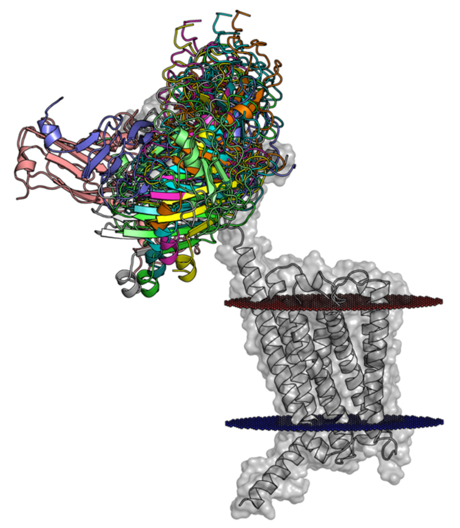
**

**Supplementary Figure 5. Schematic representation of top binding clusters solutions of CRF-BP protein-protein docking to CRF_2α_R.**

**References**

1. Wiederstein M, Sippl MJ. ProSA-web: interactive web service for the recognition of errors in three-dimensional structures of proteins. *Nucleic Acids Research* (2007) **35**(suppl 2):W407-W10. doi: 10.1093/nar/gkm290.

2. Jorgensen WL, Chandrasekhar J, Madura JD, Impey RW, Klein ML. Comparison of simple potential functions for simulating liquid water. *The Journal of Chemical Physics* (1983) **79**(2):926-35. doi: 10.1063/1.445869.

3. Lee J, Cheng X, Swails JM, Yeom MS, Eastman PK, Lemkul JA, et al. CHARMM-GUI Input Generator for NAMD, GROMACS, AMBER, OpenMM, and CHARMM/OpenMM Simulations Using the CHARMM36 Additive Force Field. *Journal of Chemical Theory and Computation* (2016) **12**(1):405-13. doi: 10.1021/acs.jctc.5b00935.
